# Prevalence of actual uptake and willingness to use pre-exposure prophylaxis to prevent HIV acquisition among men who have sex with men in Hong Kong, China

**DOI:** 10.1371/journal.pone.0191671

**Published:** 2018-02-12

**Authors:** Zixin Wang, Joseph T. F. Lau, Yuan Fang, Mary Ip, Danielle L. Gross

**Affiliations:** 1 Centre for Health Behaviours Research, JC School of Public Health and Primary Care, The Chinese University of Hong Kong, Hong Kong SAR, China; 2 Shenzhen Research Institute, The Chinese University of Hong Kong, Shenzhen, China; 3 Community Research Program on AIDS, JC School of Public Health and Primary Care, The Chinese University of Hong Kong, Hong Kong SAR, China; Chinese Academy of Medical Sciences and Peking Union Medical College, CHINA

## Abstract

**Objectives:**

This study was to investigate the prevalence of actual uptake of pre-exposure prophylaxis (PrEP), willingness to use daily oral PrEP under two cost scenarios, and potential issues related to PrEP use among men who have sex with men (MSM) with HIV negative/unknown sero-status in Hong Kong, China. Factors associated with the two measures of willingness were also investigated.

**Methods:**

403 eligible MSM completed the anonymous venue-based face-to-face interview/telephone interview.

**Results:**

Only 1% had ever used PrEP. After being briefed about some facts of PrEP, the prevalence of willingness to use daily oral PrEP was 7.7% if they could purchase PrEP at private hospitals/clinics at HK$8,000 (US$1,032)/month (market rate) and 45.2% if free PrEP was provided by public hospitals/clinics in Hong Kong (free PrEP). After adjusted for significant background variables, significant factors of these two measures of willingness included: (1) number of correct responses to knowledge on PrEP, (2) perceived risk of HIV infection in the next six months, and (3) constructs of the Theory of Planned Behavior: positive and negative attitudes toward PrEP, perceived their significant others would support them take PrEP (perceived subjective norm), perceived number of local MSM who were willing to take PrEP (perceived descriptive norm), and confidence in using PrEP under the two scenarios (perceived behavioral control). Among all participants, 9.7% and 25.1% would start and discontinue using PrEP without consulting doctors; 26.0% would not take PrEP daily if it was provided for free, and 42.4% would not take up HIV testing every 3 months after using PrEP.

**Conclusion:**

Different strategies should be considered for promoting PrEP at market rate and if free PrEP is made available. Future promotion should increase their knowledge about PrEP, modify their perceptions related to HIV and PrEP, and address some potential issues related to PrEP use.

## Introduction

Global HIV epidemic among men who have sex with men (MSM) remained uncontrolled [1]. In Hong Kong, the HIV prevalence among MSM increased to 5.85% in 2014 [2]; 79% of all reported new cases were attributed to MSM (n = 389) in first half of 2017 [3].

Pre-exposure prophylaxis (PrEP) refers to the initiation of Tenofovir Disoproxil Fomarate (TDF) / Emtricitabine (FTC) before and during periods of HIV exposure among HIV-negative individuals in order to prevent HIV acquisition [4]. With good adherence, oral PrEP could significantly reduce the risk of HIV infection among various at-risk populations [4–15]. In 2014, the World Health Organization (WHO) strongly recommended PrEP as ‘an additional HIV prevention choice within a comprehensive HIV package for MSM’ [16]. In 2016, the recommendation was expanded to include all people at substantial risk of HIV infection [17].

As of 2016, numerous countries have issued full regulatory approval for using PrEP to prevent HIV. At least four countries (i.e., France, South Africa, Kenya and Israel) are providing free PrEP [18, 19]. In the U.S., PrEP users under Medicaid or Affordable Care Act (ACA) Plans may be asked for co-payments which can be covered by other sources (e.g., Gilead Medication Assistance Plan). Some private insurances cover PrEP and some states have expended Medicaid to provide PrEP [20]. In Canada, PrEP users under the Canadian and Ontario insurance plans will be asked for some co-payments depending on their income level [21]. In Asia, Thailand and Taiwan have programs promoting PrEP use among high risk groups by partially subsidizing the cost of PrEP [22, 23]. In Hong Kong, the public hospitals/clinics are not providing PrEP to MSM. MSM can obtain PrEP legally from some private clinics at about HK$8,000 (US$ 1,032)/month. There are observations that some MSM in Hong Kong obtain PrEP from the Internet and via visits to other countries [24]. However, no medical insurance or subsidization can cover the cost of PrEP.

Data on prevalence of uptake and willingness to use PrEP among MSM is warranted to inform cost-effectiveness modeling and to facilitate related policy making [25]. Recent data showed that prevalence of PrEP use was 2.5% in Australia [26], 9.2% in the U.S. [27], and 11.0% in 15 European countries [28]. One intervention study invited 1,033 MSM in China to use free daily PrEP. The actual uptake rate was only 2.5% [29]. Prevalence of PrEP use among MSM in Hong Kong is unknown.

The Hong Kong AIDS Advisory Council strongly recommends conducting PrEP implementation studies to yield important information such as finding an appropriate delivery model [30]. In order to design effective programs, it is important to understand MSM’s willingness to use PrEP under the current situation (PrEP is provided by private hospitals/clinics at market rate) and if free PrEP is made available at public hospitals/clinics [20], as well as related barriers and facilitators. In literature, 46.1–71.0% of MSM were willing to use PrEP if it was provided for free [31–37], while such prevalence dropped to 14.0 to 36.8% if out-of-pocket payment was involved [31, 38–40]. The prevalence of willingness varied across countries, and country-specific factors were likely to affect the willingness [31–40]. We only identified three studies investigating willingness to use PrEP among MSM in China [35, 39, 41]. The prevalence of willingness was 64.0–67.8% without mentioning the cost [35, 41], 71.0% if PrEP was provided for free [35] and 23.0% if it cost US$ 340 per month [39]. However, no study reported factors associated with willingness to use free PrEP and only one reported that some positive attitudes toward PrEP (e.g., belief that PrEP would prevent HIV) were significant factors of willingness to use chargeable PrEP (US$ 340 per month) [39]. The other two studies investigated factors associated with willingness to use PrEP that was not conditioned on any cost scenarios. Significant associated factors included those related to socio-demographics, risk behaviors, prior knowledge of PrEP, and perceptions related to HIV risk and PrEP (e.g., perceived efficacy of PrEP, concerns related to side-effects) [35, 41]. These factors were considered in this study.

A meta-analysis showed that theory-based interventions are more effective than non-theory-based [42]. The Theory of Planned Behavior (TPB) [43] is a commonly used theory which postulates that behavioral intention to adopt a health-related behavior (e.g., use PrEP) is a strong predictor of actual behavior. In order to form such an intention, one would first evaluate the behavior (positive and negative attitudes), consider whether their significant others would support such behavior (perceived subjective norm), perceive how common the behavior of concern is among their peers (perceived descriptive norm), and appraise how much control they have over the behavior (perceived behavioral control) [43]. We used TPB as the framework of this study and tested significance of its constructs.

It is also important to understand some potential issues among potential PrEP users, such as non-adherence to daily dosage, not taking up HIV testing regularly, and not seeking medical consultation before they decide to start or discontinue using PrEP. These issues, as highlighted by the U.S. PrEP Clinical Practice Guideline [44] and the WHO guideline [17], should be monitored and prevented to ensure the effectiveness of PrEP and safety of PrEP users. In this study, prevalence of behavioral intention to have the aforementioned behaviors after using PrEP was also investigated.

In this study, we investigated the actual uptake of daily oral PrEP and willingness to use daily oral PrEP among MSM with HIV-negative/unknown sero-status in Hong Kong, China. Willingness to use PrEP was assessed under two scenarios, if: 1) PrEP could be obtained from local private hospitals/clinics at the market price of HK$8,000 (US$ 1,032)/month, and 2) free PrEP could be obtained from local public hospitals/clinics. We also investigated factors associated with these two measures of willingness.

## Methods

### Participants and data collection

Inclusion criteria were: 1) males who are ethnic Chinese aged 18–60 years, 2) having a Hong Kong ID card, and 3) having had anal intercourse with at least one man in the last six months. Those self-reported as HIV-positive were excluded.

A recent mapping exercise was conducted by the government and identified 12 gay bars and 16 gay saunas in Hong Kong. Upon approval of the owners, trained and experienced fieldworkers approached prospective participants in these venues at different time slots during weekdays and weekends. They briefed the prospective participants about the details and gave them an information sheet. Guarantees were made on anonymity and right to quit at any time. Verbal instead of written informed consent was obtained to maintain anonymity. The fieldworkers signed a form pledging that the participants had been fully informed about the study. A venue-based face-to-face interview was then conducted by the fieldworkers, which took about 20 minutes to complete.

In addition, participants were recruited by advertisements displayed on websites frequently visited by MSM, and referrals made by the participants. Interested participants contacted the research team by telephone. By appointment and through telephone, the fieldworkers briefed the prospective participants about details of the study, obtained their verbal informed consent, and performed the telephone interview which took about 20 minutes to complete.

A total of 567 eligible MSM were approached through outreach in gay venues (n = 323), online recruitment (n = 60) and peer referral (n = 184), and 403 of them (71.1%) provided verbal consent and completed the interview (venue: 232, online: 60, referral: 111). Upon completion of the interview, a HK$50 (US$8) supermarket coupon was given to the participants for their time spent. Ethics approval was obtained from the Survey and Behavioral Research Ethics Committee of the Chinese University of Hong Kong.

### Measures

#### Background characteristics of the participants

Information collected included socio-demographics (age, marital status, education level, and employment status), sexual orientation, utilization of HIV prevention services and sexual behaviors in the last six months (i.e., any anal intercourse with male regular or non-regular sex partners, any condomless anal sex (CAS) with men, presence of multiple male sex partnerships, and use of sexual potency drugs or illicit drugs before/during sexual intercourse). Multiple male sex partnership was defined as having had anal intercourse with at least two men in the last six months. Male regular sex partner (RP) was defined as those who were in a stable relationship and did not involve transactional sex, while male non-regular sex partner (NRP) was defined as men who were not RP and did not involve transactional sex.

#### Prevalence of actual uptake and willingness to use PrEP

Participants were asked whether they had ever used PrEP before. Participants were briefed with the following: *“PrEP is a strategy that promotes taking oral antiretroviral drugs to prevent HIV infection among HIV-negative individuals*. *PrEP is strongly recommended by the WHO as an additional HIV prevention strategy for MSM*. *You are required to take PrEP once every day when you are using it in order to achieve its effect in preventing HIV infection*. *Daily use of oral PrEP can reduce risk of HIV infection by 92%*. *PrEP has possible side effects such as nausea*, *vomiting and headache*. *PrEP can be obtained at private hospitals/clinics in Hong Kong at about HK$8*,*000 (US$ 1*,*032) per month*. *However*, *it is not yet available at governmental hospitals/clinics in Hong Kong”*. They were then asked whether they were willing to take a once-daily oral pill as PrEP for the next six months under two conditions: 1) if it could be purchased at private hospitals/clinics at HK$8,000 (US$ 1,032) per month and 2) if it could be provided for free by public hospitals/clinics in Hong Kong (Responses categories: 1 = definitely not, 2 = probably not, 3 = neutral, 4 = probably will, 5 = definitely will). Responses were then dichotomized. Willingness to use daily PrEP was defined as “probably will” or “definitely will”. Such a definition has been commonly used in previous studies [35, 39].

#### Knowledge of PrEP

Six items were used to assess knowledge of PrEP (e.g., availability of PrEP-related services in Hong Kong). A composite indicator variable was constructed by counting the number of correct responses reflecting knowledge of PrEP (ranged from 0 to 6).

#### Perceptions related to PrEP based on TPB

Five scales were constructed for this study. They were based on the TPB.

Positive attitudes toward PrEP were measured by three items. They were: 1) ‘PrEP provides you more choices for HIV prevention’, 2) ‘PrEP increases your capacity in preventing’ and 3) ‘PrEP would reduce your worry of HIV infection’. The Positive Attitude Scale was formed by summing up individual item scores (from 1 = strongly disagree to 5 = strongly agree). Higher score on the scale indicated more positive attitudes toward PrEP.

Negative attitudes toward PrEP were assessed by four items, including: 1) ‘PrEP would cause inconvenience in your daily life’, 2) ‘long-term usage of PrEP would cause severe harm on your physical health’, 3) ‘others would think you are having high risk behaviors if you are using PrEP’ and 4) “you will be stigmatized by medical professionals when you using PrEP-related services’. The Negative Attitude Scale was formed by summing up individual item scores (from 1 = strongly disagree to 5 = strongly agree). Higher score on the scale indicated more negative attitudes toward PrEP.

Two items (from 1 = strongly disagree to 5 = strongly agree) were used to measure participants’ perceived support from their significant others (people who are important to them and their male sex partners) for their PrEP use. The Perceived Subjective Norm Scale was constructed by summing up individual item scores. Higher score indicated perceived subjective norm more supportive of PrEP use.

Two items (from 1 = strongly disagree to 5 = strongly agree) were used to measure participants’ perceived behavioral control in obtaining PrEP and taking PrEP every day if they can purchase it from private clinics. Two similar items were used to measure participants’ perceived behavioral control of using free PrEP. Two scales (the Perceived Behavioral Control of Using Self-purchased PrEP Scale and the Perceived Behavioral Control of Using Free PrEP Scale) were constructed by summing up individual item scores. Higher score indicated higher perceived behavioral control in using PrEP under the two scenarios. Cronbach’s alpha for these scales ranged from 0.654 to 0.853.

In addition, one item was used to measure perceived level of financial burden of using daily PrEP at a cost of HK$8,000 per month (response categories: 1 = very low, 5 = very high), another negative attitude toward PrEP. Another single item was used to measure perceived descriptive norm related to PrEP (i.e., ‘how many MSM in Hong Kong are willing to take PrEP to prevent HIV infection?’ response categories: 1 = none, 6 = great many).

#### Perceptions related to HIV risk

Participants were also asked about their perceived risk of HIV infection in the next six months under their current situation (i.e., physical conditions and sexual behaviors). (Response categories: 1 = very low, 5 = very high).

#### Potential issues related to PrEP use

Participants were asked whether they would: 1) start using PrEP without consulting doctors if it could be purchased online, 2) not take PrEP once-daily if it was provided for free, 3) not take up HIV testing every 3 months after using PrEP, and 4) discontinue using PrEP without consulting doctors (Response categories: 1 = definitely not, 5 = definitely will).

### Statistical analysis

Using the two measures of willingness to use PrEP as dependent variables, univariate odds ratios (ORu) for the associations between background independent variables and the dependent variables were estimated. Those background variables with p<0.10 in the univariate analysis were adjusted for in the subsequent multiple logistic regression analysis involving other independent variables (i.e., knowledge of PrEP, perceptions related to HIV risk and PrEP). Adjusted odds ratios (AOR) and respective 95% confidence interval (CI) were derived from such analyses. SPSS version 16.0 was used for data analysis, with p values <0.05 taken as statistically significant.

## Results

### Background characteristics

Majority of the participants were 18 to 30 years old (66.0%), were currently single (77.9%), had attained college education or above (80.6%), had had a full-time job (77.7%), and identified themselves as homosexuals (87.6%). About half of the participants had taken up HIV testing (51.6%) or other HIV-related prevention services (44.9%) in the last six months. In the last six months, 81.1% and 58.1% had had anal intercourse with RP and NRP, 58.3% and 39.7% reported multiple male sex partnerships and CAS with men, respectively, and 7.4% and 3.7% reported use of sexual potency drugs and illicit drugs before/during sexual intercourse, respectively. (Table 1)

**Table 1:** Background characteristics of men who have sex with men (n = 403).

	n	%
**Socio-demographics**		
Age group		
18–25	131	32.5
26–30	135	33.5
31–35	52	12.9
>35	85	21.1
Marital status		
Currently single	314	77.9
Cohabitate / married with men	85	21.1
Cohabitate / married with women	4	1.0
Education level		
Secondary or below	78	19.4
College or above	325	80.6
Employment status		
Full-time	313	77.7
Part-time / unemployed / retired / student	90	22.3
**Sex-related information**		
Sexual orientation		
Homosexual	353	87.6
Bisexual	50	12.4
**Utilization of HIV-related prevention services in the last six months**		
HIV testing		
No	195	48.4
Yes	208	51.6
Other HIV-related prevention services (e.g., receiving condoms, peer education, or education pamphlets)		
No	222	55.1
Yes	181	44.9
**Sexual behaviors in the last six months**		
Had had anal intercourse with regular male sex partner(s) (RP)		
No	76	18.9
Yes	327	81.1
Had had anal intercourse with non-regular male sex partner(s) (NRP)		
No	169	41.9
Yes	264	58.1
Multiple male sex partnerships		
No	168	41.7
Yes	265	58.3
Condomless anal sex (CAS) with men		
No	243	60.3
Yes	160	39.7
Use of illicit substances before / during sexual intercourse (Chemsex)		
No	388	96.3
Yes	15	3.7
Use of sexual potency drugs before / during sexual intercourse		
No	373	92.6
Yes	30	7.4

### Prevalence of use and willingness to use PrEP

Only four participants (1.0%) reported that they had used PrEP before. After being briefed about some facts of PrEP as mentioned in the Method, the prevalence of willingness to use daily oral PrEP was 7.7% if they could purchase PrEP at private hospitals/clinics at HK$8,000 (US$1,032)/month and 45.2% if free PrEP was provided by public hospitals/clinics in Hong Kong. (Table 2)

**Table 2:** Descriptive statistics of dependent and independent variables (n = 403).

	n	%	Mean scale score (SD^1^, range)
**Utilization of PrEP**			
Had ever used PrEP			
No	399	99.0	
Yes	4	1.0	
**Knowledge on PrEP**			
Availability of PrEP-related services in Hong Kong			
No	5	1.2	
Yes*	30	7.4	
Never heard of PrEP / uncertain	368	91.4	
People can obtain PrEP without doctors’ prescriptions in Hong Kong			
No*	41	10.2	
Yes	3	0.7	
Never heard of PrEP / uncertain	359	89.1	
People can stop using PrEP without asking doctors’ opinion			
No*	48	11.9	
Yes	10	2.5	
Never heard of PrEP / uncertain	345	85.6	
Number of tablets required to take every day to prevent HIV			
One*	33	8.2	
More than one	8	2.0	
Never heard of PrEP / uncertain	362	89.8	
Whether PrEP can prevent sexually transmitted diseases (e.g., syphilis, genital warts)			
No*	52	12.9	
Yes	7	1.7	
Never heard of PrEP / uncertain	344	85.4	
Whether WHO recommends MSM to use PrEP for HIV prevention			
No	20	5.0	
Yes*	17	4.2	
Never heard of PrEP / uncertain	366	90.8	
Number of correct responses			
0	325	80.6	
1–3	54	13.4	
4–6	24	6.0	
**Willingness to use daily oral PrEP in the next six months under different scenarios after being briefed about facts of PrEP**			
Willing to take once-daily oral pill as PrEP in the next six months if PrEP could be purchased at private hospitals/clinics at HK$8,000 (US$ 1,032) per month			
No (definitely not / probably not / neutral)	372	92.3	
Yes (probably will / definitely will)	31	7.7	
Willing to take once-daily oral pill as PrEP in the next six months if free PrEP could be provided by public hospitals/clinics in Hong Kong			
No (definitely not / probably not / neutral)	221	54.8	
Yes (probably will / definitely will)	182	45.2	
**Cognitive variables related to PrEP based on TPB**			
Positive attitudes towards PrEP (% agree / strongly agree)			
PrEP provides you more choice for HIV prevention	197	48.9	
PrEP increases your capacity in preventing HIV	265	65.8	
PrEP would reduce your worry of HIV infection	229	56.8	
*Positive Attitude Scale*			10.3 (1.7, 6–12)
Negative attitudes towards PrEP (% agree / strongly agree)			
PrEP would cause inconvenience in your daily life	153	37.2	
Long-term usage of PrEP would cause severe harm on your physical health	189	46.9	
Others would think you are having high risk behaviors if you are using PrEP	148	36.7	
You will be stigmatized by medical professionals when you are using PrEP-related services	125	31.0	
*Negative Attitude Scale*			12.5 (2.4, 6–18)
Perceived level of financial burden caused by daily use of PrEP			
Very low / low / moderate	71	17.6	
High / very high	332	82.4	
Subjective norm related to PrEP (% agree / strongly agree)			
People who are important to you will support you take PrEP	159	39.5	
Your male sex partners will support you take PrEP	120	29.8	
*Subjective Norm Scale*			6.1 (2.0, 2–10)
Descriptive norm related to PrEP			
How many MSM in Hong Kong who are willing to take PrEP to prevent HIV infection?			
None / very few / few	207	51.4	
Some / many / great many	196	48.6	
Perceived behavioral control of using self-purchased PrEP (% agree / strongly agree)			
You are confident in obtaining PrEP in the next six months if you can purchase it from private clinics at HK$8,000 per month	16	4.0	
You are confident in taking PrEP every day in the next six months if you can purchase it from private clinics at HK$8,000 per month	36	8.9	
*Perceived Behavioral Control of Using Self-purchased PrEP Scale*			3.4 (1.8, 2–10)
Perceived behavioral control of using free PrEP (% agree / strongly agree)			
You are confident in obtaining PrEP in the next six months if it is available for free in governmental hospitals or clinics	129	32.0	
You are confident in taking PrEP every day in the next six months if it is available for free in governmental hospitals or clinics	124	30.8	
*Perceived Behavioral Control of Using Free PrEP Scale*			5.8 (2.3, 2–10)
**Perception related to HIV risk**			
Perceived risk of HIV infection in the next six months under your current situation (i.e., physical conditions and sexual behaviors)			
Very low / low	335	83.1	
Moderate / high / very high	68	16.9	

* Correct response

SD: standard deviation

### Knowledge of PrEP and perceptions related to HIV risk and PrEP

The prevalence of correct responses for the individual knowledge items related to PrEP ranged from 4.2% (WHO recommends MSM to use PrEP for HIV prevention) to 12.9% (PrEP cannot prevent sexually transmitted diseases such as syphilis and genital warts). Item responses and means (standard deviation, SD) of the scales related to perceptions of HIV and PrEP were described in Table 2.

### Factors associated with willingness to use daily oral PrEP at 8,000 HKD/month

In the univariate analysis, two background variables (sexual orientation and had had anal intercourse with RP) were significantly associated with this measure of willingness. After adjusting for these two variables, the Subjective Norm Scale (AOR: 1.44, 95%CI: 1.16, 1.78) and Perceived Behavioral Control of Using Self-purchased PrEP Scale (AOR: 1.56, 95%CI: 1.30, 1.87) were positively associated with this measure of willingness, while a negative association was found for the perceived level of financial burden caused by daily use of PrEP (AOR: 0.42, 95%CI: 0.18. 0.95). (Tables 3 and 4).

**Table 3:** Background variables associated with two measures of willingness to use daily oral PrEP (n = 403).

	Willing to take once-daily oral pill as PrEP if it could be purchased at private hospitals/clinics at HK$8,000 / month	Willing to take once-daily oral pill as PrEP if free PrEP could be provided by public hospitals/clinics in Hong Kong
	ORu (95%CI)	ORu (95%CI)
**Social-demographics**		
Age group		
18–25	1.0	1.0
26–30	0.97 (0.42, 2.24)	0.64 (0.39, 1.03)†
31–35	0.83 (0.25, 2.69)	0.45 (0.23, 0.87)*
>35	0.36 (0.10, 1.33)	0.59 (0.34, 1.03)†
Marital status		
Currently single	1.0	1.0
Cohabitate / married with men	0.92 (0.36, 2.32)	0.87 (0.53, 1.41)
Cohabitate / married with women	4.03 (0.40, 40.22)	1.18 (0.16, 8.49)
Education level		
Secondary or below	1.0	1.0
College or above	1.68 (0.57, 4.94)	1.15 (0.70, 1.90)
Employment status		
Full-time	1.0	1.0
Part-time / unemployed / retired / student	1.02 (0.42, 2.44)	1.53 (0.95, 2.45)†
**Sex-related information**		
Sexual orientation		
Homosexual	1.0	1.0
Bisexual	2.73 (1.15, 6.50)*	1.25 (0.69, 2.26)
**Utilization of HIV-related prevention services in the last six months**		
HIV testing		
No	1.0	1.0
Yes	1.00 (0.48, 2.08)	0.85 (0.58, 1.27)
Other HIV-related prevention services (e.g., receiving condoms, peer education, or education pamphlets)		
No	1.0	1.0
Yes	0.56 (0.26, 1.22)	1.24 (0.83, 1.84)
**Sexual behaviors in the last six months**		
Had had anal intercourse with regular male sex partner(s) (RP)		
No	1.0	1.0
Yes	0.39 (0.18, 0.84)*	1.09 (0.66, 1.80)
Had had anal intercourse with non-regular male sex partner(s) (NRP)		
No	1.0	1.0
Yes	1.16 (0.55, 2.45)	1.30 (0.87, 1.94)
Multiple male sex partnerships		
No	1.0	1.0
Yes	0.75 (0.36, 1.55)	1.22 (0.82, 1.82)
CAS with men		
No	1.0	1.0
Yes	0.71 (0.32, 1.54)	1.64 (1.09, 2.45)*
Use of illicit substances before / during sexual intercourse (Chemsex)		
No	1.0	1.0
Yes	1.91 (0.41, 8.85)	1.86 (0.65, 5.34)
Use of sexual potency drugs before / during sexual intercourse		
No	1.0	1.0
Yes	0.85 (0.19, 3.74)	3.07 (1.37, 6.89)***

ORu: univariate odds ratios

† P<0.10

* P<0.05

*** P<0.001

**Table 4:** Factors associated with two measures of willingness to use daily oral PrEP (n = 403).

	Willing to take once-daily oral pill as PrEP if it could be purchased at private hospitals/clinics at HK$8,000 / month		Willing to take once-daily oral pill as PrEP if free PrEP could be provided by public hospitals/clinics in Hong Kong	
	ORu (95%CI)	AOR (95%CI)	ORu (95%CI)	AOR (95%CI)
**Knowledge on PrEP**				
Number of correct responses				
0	1.0	1.0	1.0	1.0
1–3	2.16 (0.87, 5.35)†	2.50 (0.98, 6.36)†	2.42 (1.34, 4.39)**	2.39 (1.30, 4.39)**
4–6	2.07 (0.57, 7.50)	2.45 (0.65, 9.27)	2.00 (0.86, 4.63)	1.50 (0.62, 3.66)
**Cognitive variables related to PrEP based on TPB**				
Positive Attitude Scale	0.91 (0.74, 1.11)	**---**	1.26 (1.11, 1.42)***	1.23 (1.09, 1.40)**
Negative Attitude Scale	0.92 (0.79, 1.07)	**---**	0.79 (0.72, 0.86)***	0.78 (0.71, 0.85)***
Level of financial burden caused by daily use of PrEP				
Very low/low/moderate	1.0	1.0		
High/very high	0.41 (0.19, 0.92)*	0.42 (0.18, 0.95)*	**---**	**---**
Subjective Norm Scale	1.47 (1.19, 1.81)***	1.44 (1.16, 1.78)**	1.58 (1.39, 1.79)***	1.60 (1.41, 1.82)***
Descriptive norm related to PrEP				
Number of MSM who are willing to take PrEP to prevent HIV infection				
None / very few / few	1.0		1.0	1.0
Some / many / great many	1.14 (0.55, 2.37)	**---**	2.12 (1.42, 3.15)***	2.08 (1.38, 3.14)***
Perceived Behavioral Control of Using Self-purchased PrEP Scale	1.51 (1.27, 1.80)***	1.56 (1.30, 1.87)***	---	**---**
Perceived Behavioral Control of Using Free PrEP Scale	**---**	**---**	1.53 (1.38, 1.71)***	1.55 (1.38, 1.73)***
**HIV risk perception**				
Perceived risk of HIV infection in the next six months under your current situation (i.e., physical conditions and sexual behaviors)				
Very low / low	1.0		1.0	1.0
Moderate / high / very high	1.81 (0.77, 4.23)	**---**	2.09 (1.23, 3.56)***	2.06 (1.18, 3.58)*

ORu: univariate odds ratios

AOR: Adjusted Odds Ratios, odds ratios adjusted by background variables with p< .10 listed in Table 3

† P < 0.10

* P < 0.05

** P < 0.01

*** P < 0.001

---: not considered in the model

### Factors associated with willingness to use free daily oral PrEP

In the univariate analysis, four background variables (age group, employment status, CAS with men and use of sexual potency drugs before/during anal intercourse) were significantly or marginally associated with this measure of willingness (ORu were shown in Table 3). Adjusting for these four background variables, all five constructs of TPB were significantly associated with this measure of willingness and the associations were in the expected directions. They were: 1) the Positive Attitude Scale (AOR: 1.23, 95%CI: 1.09, 1.40), 2) the Negative Attitude Scale (AOR: 0.78, 95%CI: 0.71, 0.85), 3) the Subjective Norm Scale (AOR: 1.60, 95%CI: 1.41, 1.82), 4) the Perceived Behavioral Control of Using Free PrEP Scale (AOR: 1.55, 95%CI: 1.38, 1.73) and 5) perceived number of local MSM who were willing to take PrEP to prevent HIV infection (perceived descriptive norm) (some/many/great many: AOR: 2.08, 95%CI: 1.38, 3.14; reference group: none/very few/few). In addition, the number of correct responses to knowledge on PrEP (1–3 correct responses: AOR: 2.39, 95%CI: 1.30, 4.39; reference group: 0 correct response), and having at least a moderate perceived risk of HIV infection in the next six months under one’s current situation (AOR: 2.06, 95%CI: 1.18, 3.58) were also significantly and positively associated with this measure of willingness (Tables 3 and 4).

### Potential issues related to PrEP use

Among all participants, 9.7% and 25.1% would definitely/probably start and discontinue using PrEP without consulting doctors, respectively. Over one quarter of them (26.0%) would definitely/probably not take PrEP daily if it was provided for free, while 42.4% would definitely/probably not take up HIV testing every 3 months after using PrEP (data not tabulated).

## Discussion

PrEP is a potential important approach to reduce the risk of HIV infection among local MSM. To our knowledge, this is the first study investigating prevalence of use of PrEP among MSM in China. Only 1% of the participants self-reported that they had ever used PrEP, such prevalence was lower than that reported in western countries [38, 45]. A mathematical model suggested that provision of PrEP to 80% of the HIV-negative MSM would achieve a 43% reduction in overall HIV incidence in this population [25]. Therefore, effective means to increase coverage of PrEP are greatly needed.

In this study, only 7.7% of the participants expressed willingness to use PrEP if it could be obtained at market rate by private clinics/hospitals. Improvements are greatly needed. Such prevalence of willingness was much lower than that reported in Taiwan (23.0%) [39] or Thailand (58.8%) [40] also based on market rate. Much higher costs of PrEP in Hong Kong (US$ 1,032/month) than Taiwan or Thailand (US$15-340/month) may partially explain the difference [39, 40]. The prevalence of willingness increased to 45.2% if free PrEP could be obtained from public hospitals/clinics. However, such prevalence was lower than that reported in western countries and mainland China [31–37]. Since only 43–62% of those with a behavioral intention would translate it into related action [46], effective health promotions are needed even if free PrEP is made available.

Our results suggests that increasing knowledge of PrEP, creating a subjective norm supporting PrEP use and enhancing perceived behavioral control of using PrEP are potentially useful to promote PrEP at market rate and after free PrEP becomes available, as these factors were significantly associated with both measures of willingness. Future health promotion should enhance of their knowledge of PrEP, encourage MSM to discuss PrEP with their significant others, so as to obtain support regarding PrEP use. Multiple strategies may be applied in future PrEP promotion programs, which may include simplification of the procedures to obtain PrEP and provision of gay-friendly PrEP-related services.

Our findings also suggested that different factors should be considered when promoting PrEP at market rate and if free PrEP is made available. Specifically, to promote PrEP at market rate, special attention should be given to those who self-identify as bisexual and those without RP, as they had higher motivation to use PrEP. Perceived high financial burden caused by daily use of PrEP was a significant factor negatively associated with this measure of willingness. Since generic lamivudine/tenofovir is available at low cost (i.e., US$57/year in the U.S.) [47] and the current patent for Truvada will expire in 2018 [47], it is expected that its market rate will drop in the future. Therefore, the financial burden caused by daily use of PrEP may not be a major issue in rolling out PrEP in future. Contrary to expectation, increasing perceived risk of HIV infection or positive attitudes toward PrEP, or reducing negative attitudes toward PrEP might not be useful in promoting PrEP under the current situation, as they were non-significant factors.

If free PrEP is made available, younger MSM may be more responsive to PrEP promotion, as they were more willing to use free PrEP. This is understandable as younger people are more receptive to innovations [48]. Promotion of free PrEP among different age groups may consider different strategies. Special attention should be given to those MSM who have CAS with men or use sexual potency drugs before/during sexual intercourse, as they showed higher willingness to use free PrEP. They might have perceived a stronger need for taking protective measures against HIV. Health promotion should also increase their positive attitudes and reduce their negative attitudes toward PrEP. Health communication messages should emphasize PrEP as an effective approach to increase ability of HIV prevention and reduce psychological burden related to HIV, PrEP is safe in long-term (e.g., severe side effects are rare) and common side effects (e.g., nausea, vomiting and headache) can be easily managed [49]. Positive experiences shared by peer PrEP users may be useful to remove concerns about inconvenience in daily life caused by PrEP. Previous studies showed that PrEP-related stigma was an important barrier hindering people at risk of HIV to take up and adhere to PrEP [50]. Health education should be conducted not only among MSM, but also among general public and healthcare professionals to increase their understanding of PrEP. PrEP should be seen as a health option and a socially acceptable strategy for HIV prevention. Perceived higher proportion of MSM who are willing to take PrEP was also associated with higher willingness. New norms about willingness need to be built. The Diffusion of Innovation Theory is applicable to this case, which states that according to the length of innovation-decision process, people can be divided into innovators, early adopters, late majority and the laggards [51]. It is important to identify a group of innovators (i.e., PrEP users) in the MSM community. These innovators can help import this new behavioral into the MSM community and their positive experiences would draw the attention of early adopters. Testimonials from innovators and early adopters are potentially useful to establish new norm supporting PrEP use among MSM. Websites/forums frequently visited by MSM and gay social networking smartphone applications (e.g., Grindr) are potential suitable platforms to publicize such testimonials. Moreover, despite their high risk profiles, only a few of them perceived a moderate or higher risk of HIV infection. In our study, perceived risk of HIV infection was associated with higher willingness to use PrEP. It is important for future free PrEP promotion campaigns to increase HIV risk perception among MSM. In addition to these strategies, there are other potential barriers and facilitators for increasing PrEP uptake when free PrEP is provided by public hospitals/clinics. Frist of all, public hospitals/clinics in Hong Kong are often criticized for its long waiting period to see a specialist [52], it is important to shorten the waiting period for MSM to obtain PrEP in future. Moreover, local public hospitals/clinics should consider providing a free service package for PrEP users, including HIV, other sexually transmitted diseases, liver/kidney function testing, sexual health care, and adherence support. Free access to these services was a facilitator for PrEP uptake in western countries [53].

In addition, future PrEP promotion campaigns should also address potential issues identified by this study. Informal PrEP use without prescription was observed in the Netherlands and U.K. [54]. It is also likely to happen among MSM in Hong Kong, as 9.7% would start PrEP without consulting doctors. Moreover, 25.1% of them would discontinue PrEP without medical consultation. Health communication messages should emphasis the importance of medical consultation when one decides to start or discontinue PrEP. Counseling supporting adherence is also needed, as about a quarter of them did not intend to adhere to daily dosage. Regular uptake of HIV testing (e.g., every three months) is essential for PrEP users, as it can allow for early HIV diagnosis, counseling for those with acute HIV infection, and a minimized risk of drug resistance [17]. However, about half of them did not intend to do so. They should be reminded about the importance of regular HIV testing when using PrEP. Future studies should understand reasons behind such behavioral intentions.

This study has some limitations. First, similar to previous studies investigating willingness to use PrEP among MSM; recruitment was not based on random sampling. The results may not be representative of MSM in China, and caution should be taken when generalizing the results to this population in China. Second, we did not ask prior awareness of PrEP. Although we have explained benefits and harms before asking about willingness to ensure that uniform information has been received by all participants, first time knowledge of PrEP may lower the reliability of willingness. Moreover, we did not use validated scales measuring concerns about using PrEP and willingness to use PrEP [55]. The question items were constructed for this study. Furthermore, given the low HIV testing rate among MSM in Hong Kong, this study might have included some HIV-positive MSM. Selection bias might exist. Lastly, this was a cross-sectional study that could not establish causality.

## Conclusions

PrEP is a potential important strategy in reducing HIV incidence among MSM in Hong Kong. Prevalence of PrEP use is very low among this group, effective strategies to increase PrEP use should be considered. Different strategies should be considered for promoting PrEP at market rate and if free PrEP is made available. Future promotion should increase their knowledge about PrEP, modify their perceptions related to HIV and PrEP, and address some potential issues related to PrEP use.

## Research involving human participants

All procedures performed in studies involving human participants were in accordance with the ethical standards of the Survey and Behavioural Research Ethics Committee, the Chinese University of Hong Kong and with the 1964 Helsinki declaration and its later amendments or comparable ethical standards.

## Informed consent

Informed consent was obtained from all individual participants included in the study.
